# The Story of the Insane from Year to Year

**Published:** 1900-09-22

**Authors:** 


### THE STORY OF THE INSANE FROM YEAR TO YEAR.

(Twelfth Series.)

### THE SOMERSET COUNTY ASYLUM AT WELLS.

On January 1st, 1898, this asylum contained 800 patients, and on December 31st the numbers had increased by 27. There were 276 admissions, 28 readmissions, 76 recoveries, 16 relieved, and 70 deaths. The recoveries stand at 34·8 per cent. of the admissions, and the deaths at 8·9 per cent. of the average number resident. Of the year's deaths, 19 were caused by cerebral and spinal diseases, 5 by pneumonia, and 6 by consumption. Of the year's admissions, 28 owed their insanity to various moral causes, 24 to intemperance in drink, 41 to hereditary influence, and in 54 no cause was known. Although the opening of the second asylum for this county is only a matter of yesterday, Dr. Wade states that there are only 13 vacant beds on the male side of his asylum, and that further accommodation is required, as the new asylum is practically full. It is sometimes said that asylum nurses cannot have much experience in surgical nursing, and no doubt this is so; but opportunities are never wholly absent. At the Somerset Asylum the following surgical cases were under treatment: Fracture of the femur, trephining of skull, cancer of breast, lithotomy, intra-abdominal abscess, and abdominal section. The committee have under consideration the improvement of the asylum. It is proposed to build a new house for the medical superintendent, to add to and alter some of the wards, and to erect a small hospital for infectious disease. It is to be hoped that the latter will not be for less than 20 patients. If so it is likely to prove a snare and a delusion. The weekly cost of maintenance was 8s. 9d. per head.

### THE SOMERSET COUNTY ASYLUM AT COTFORD.

The second annual report shows that 313 patients were in residence at the beginning of the year 1898, and that the number had increased to 459 by the end of the year. There were 199 first admissions, 5 readmissions, 26 recoveries, 7 discharged relieved, and 24 deaths. Doubtless owing to the admission of a great many chronic cases the percentage of recoveries is only 12·74 on the admissions. The deaths stand at 5·48 per cent. on the average number resident. Of the deaths 7 were caused by cerebral and spinal diseases, 2 by consumption, 1 by pneumonia, and 1 by enteritis. Table X tells us that in 20 of the year's admissions the insanity was caused by moral causes, in 10 by drink, and in 20 by hereditary influences. Although only opened two years since the asylum is full on the men's side, and further provision is already needed to keep pace with the influx of fresh admissions. It is not easy to understand why the accommodation was not larger to begin with. In this division of Somerset 70 per cent. of the pauper insane are in the asylum, 10 per cent. are in workhouses, and 20 per cent. are boarded out or living with their friends. It will not be easy to increase the two latter percentages, and hence the burden must fall on the asylum. The weekly rate was 9s. 4½d. per head, a not unsatisfactory amount for so new an asylum.

